# *RAPSN*-Associated Congenital Myasthenic Syndrome due to Biallelic Single Nucleotide Variants at the Same Position

**DOI:** 10.1155/crig/1882021

**Published:** 2025-10-20

**Authors:** Laura Keehan, Jennefer N. Carter, Elijah Kravets, Matthew T. Wheeler, Jonathan A. Bernstein, Ricardo A. Maselli, Jacinda B. Sampson, Suha Bachir

**Affiliations:** ^1^Department of Pediatrics, Division of Medical Genetics, Stanford University School of Medicine, Palo Alto 94304, California, USA; ^2^Stanford Center for Undiagnosed Diseases, Stanford University, Stanford 94305, California, USA; ^3^Division of Cardiovascular Medicine, Stanford University School of Medicine, Stanford 94305, California, USA; ^4^Department of Neurology, University of California Davis, Sacramento, California, USA; ^5^Department of Neurology, Stanford University School of Medicine, Stanford 94305, California, USA

**Keywords:** congenital myasthenic syndrome, genome sequencing, multiallelic variant, p.(N88K), *RAPSN*, rapsyn

## Abstract

Biallelic pathogenic variants in *RAPSN* cause a form of congenital myasthenic syndrome (CMS), which is typically characterized by fatiguable muscle weakness, hypotonia, and feeding difficulties that present in the neonatal period or early childhood. *RAPSN*-associated CMS can be treated with acetylcholinesterase inhibitors. Here, we present a 4-year-old male with a history of neonatal respiratory distress, hypotonia, and muscle weakness exacerbated by illness who underwent trio genome sequencing and was found to have biallelic single nucleotide variants at the same position in *RAPSN*, encoding NM_005055.5:c.264C > A p.(N88K) and NM_005055.5:c.264C > G p.(N88K). The paternally inherited c.264C > G variant has not been previously reported. Interestingly, only the maternally inherited c.264C > A variant was reported on the patient's prior clinical exome sequencing, which delayed diagnosis and initiation of treatment for this patient. This case highlights the complexity of identifying multiallelic variants during exome and genome sequencing analysis. Additionally, this case is the first report of facial malformations in a patient with *RAPSN*-associated CMS due to variants outside of the promoter region.

**Trial Registration:** ClinicalTrials.gov identifier: NCT02450851

## 1. Introduction

Congenital myasthenic syndromes (CMSs) are a diverse group of neuromuscular disorders with genotypic and phenotypic heterogeneity that are characterized by dysfunction in neuromuscular transmission at the level of the neuromuscular junction (NMJ). Currently, pathogenic variants in 35 genes have been identified to cause autosomal dominant or autosomal recessive CMS [1]. CMS presents clinically with fatigable weakness in the ocular, bulbar, facial, respiratory, or limb muscles that can be permanent. Affected individuals usually have typical cognitive development; however, the phenotypic spectrum and age of onset can vary significantly depending on the genotype. In infants, symptoms can include joint contractures and difficulty feeding due to poor suck. In childhood onset CMS, individuals can also have scoliosis [1–4].

Pathogenic variants in the gene *RAPSN* that codes for receptor-associated protein of the synapse (rapsyn) cause CMS in an autosomal recessive manner. Rapsyn is a protein that functions to cluster and anchor acetylcholine receptors (AChR) in the postsynaptic membrane of the motor endplate within the NMJ. Therefore, variants that affect the function of rapsyn cause NMJ endplate AChR deficiency [1, 5–7]. *RAPSN*-associated CMS (OMIM #616326) can present as an early onset condition with neonatal hypotonia, respiratory failure, episodic apnea, and arthrogryposis multiplex congenita. Later onset manifestations are characterized by limb weakness in adolescence or adulthood. Additionally, pathogenic variants in *RAPSN* have been detected prenatally in fetuses affected by multiple pterygium syndrome, fetal akinesia, and nonimmune hydrops fetalis (OMIM #618388) [8–10]. Because rapsyn functions in the postsynaptic membrane of the NMJ, individuals with *RAPSN*-associated CMS typically respond to treatment with acetylcholinesterase (AChE) inhibitors [1, 2, 11, 12]. Here, we present the case of a 4-year-old male with neonatal onset hypotonia, contractures of the hands and feet, fatigable muscle weakness, ptosis, and mild dysmorphic features who was found to have biparentally inherited compound heterozygous variants at the same nucleotide position in *RAPSN*.

## 2. Methods

### 2.1. Clinical Data Collection

The patient was enrolled in the Undiagnosed Diseases Network (UDN) protocol. The study was approved by the NIH Institutional Review Board, and both parents provided written informed consent for publication of clinical information and patient photos. Phenotypic data were collected from medical records and clinical evaluation.

### 2.2. Genome Sequencing (GS)

GS was performed on genomic DNA from patient and parental blood specimens. Data analysis and interpretation were performed by the Baylor Genetics analytics pipeline as previously described [13]. The variants were interpreted according to American College of Medical Genetics (ACMG) guidelines. Variants were orthogonally confirmed by Sanger sequencing.

## 3. Case Presentation

The patient was born full term via urgent c-section due to failure to progress and fetal intolerance to labor. Delivery was complicated by loose nuchal cord and thick meconium. Apgar scores were 3, 6, and 8 at 1, 5, and 10 min of life, respectively. He required continuous positive airway pressure (CPAP) for the first 24 h of life due to respiratory distress. On initial exam, he was noted to have micrognathia, high arched palate, contractures of the hands and feet, low set ears, hypotonia, and poor suck. He was admitted to the neonatal intensive care unit (NICU) for the first 10 days of life for respiratory support and nutritional management with nasogastric tube due to poor feeding. Evaluation during that time included a normal eye exam and brain magnetic resonance imaging (MRI) with diffuse thinning of the corpus callosum.

**Figure 1:**
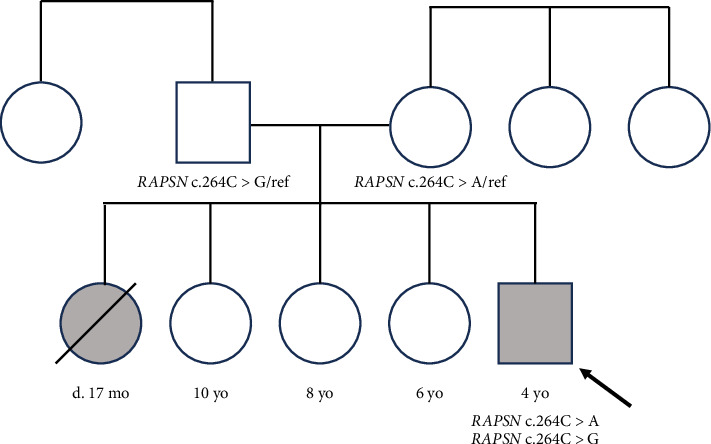
Pedigree of the patient's family. Patient indicated by arrow. Shaded shapes indicate family members with muscle weakness. Identified *RAPSN* variants written underneath individuals who had genetic testing completed. “Ref” indicates the reference allele.

**Figure 2:**
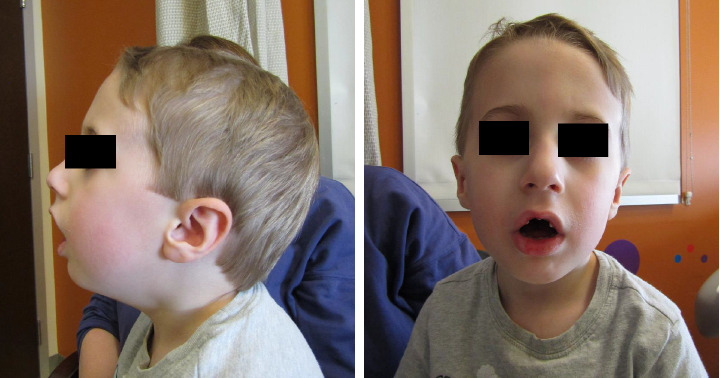
Patient images at age 4 years old demonstrating slight micrognathia and elongated face.

The patient was most recently evaluated at 4 years of age. Prior to evaluation, he had demonstrated pronounced hypotonia and weakness that was nonprogressive, and he experienced episodic exacerbations of more profound weakness during acute illnesses. He was hospitalized several times for respiratory distress requiring intubation in the setting of viral illnesses, and he had difficulty eating and drinking during these illnesses. In between these acute illnesses, he experienced cycles of sweating. From a developmental perspective, he has met motor and language milestones on time but required physical and occupational therapy due to hypotonia. He has not had any developmental regression, and there are no concerns for intellectual disability or learning disabilities. Family history is notable for a full sister who had muscle weakness and passed away at age 17 months after a suspected aspiration event and three full siblings without similar symptoms (Figure 1). Physical exam was notable for slight micrognathia, tented open mouth, bilateral ptosis, narrow and elongated face, low appendicular tone, subjectively thin and long appearing fingers, pes planus, and slight distal interphalangeal joint hypermobility (Figure 2).

From a genetic testing perspective, he had a normal chromosomal microarray and negative targeted testing for spinal muscular atrophy, myotonic dystrophy type 1, and Prader–Willi syndrome. Biochemical screening labs were within normal limits. Trio exome sequencing (ES) was overall nondiagnostic but revealed a heterozygous paternally inherited pathogenic variant in *COL6A1* c.532G > T (p.Glu178^∗^), compound heterozygous variants in *MLYCD* classified as pathogenic and VUS, respectively, c.674del (p.Met225Argfs^∗^3) and duplication of exons 2-5, a single, maternally inherited heterozygous likely pathogenic variant in *RAPSN* c.264C > A (p.N88K), two variants of uncertain significance in *DNAH11* c.7980A > C (p.Gln2660His) and c.7472G > C (p.Arg2491Pro), one variant of uncertain significance in *SOS2* c.500G > A (p.Cys167Tyr), and one variant of uncertain significance in *SUCLG1* c.24C > A. Mitochondrial DNA sequencing and deletion analysis were also nondiagnostic and revealed a variant of uncertain significance in *MT-CO1* m.6489C > A. The *COL6A1* variant can be associated with autosomal dominant inheritance but was considered unlikely to be causing his phenotype given that it was inherited from his unaffected father. The *MLYCD* and *SUCLG1* variants are associated with autosomal recessive inheritance and were considered unlikely to be causing clinical disease given he had no increase in malonic acid or methylmalonic acid on urine organic acid analysis. Variants in *DNAH11* are associated with autosomal recessive primary ciliary dyskinesia, variants in *SOS2* are associated with autosomal dominant Noonan syndrome, and variants in *MT-CO1* are associated with nonsyndromic hearing loss. Given the limited phenotypic overlap, the variants in *DNAH11*, *SOS2*, and *MT-CO1* were considered unlikely to be contributing to his presentation.

**Figure 3:**
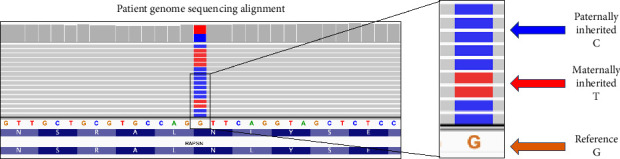
Patient genome sequencing alignment demonstrating biallelic variants in *RAPSN* at the same genomic coordinate.

The patient was referred to the UDN for further evaluation. Trio GS revealed compound heterozygous, biparentally inherited, missense variants in *RAPSN* NM_005055.5:c.264C > A p.(N88K) and NM_005055.5:c.264C > G p.(N88K) at the same nucleic acid position (GRCh38)chr11:47448079 (Figure 3). The paternally inherited variant and inheritance were confirmed by Sanger sequencing.

**Figure 4:**
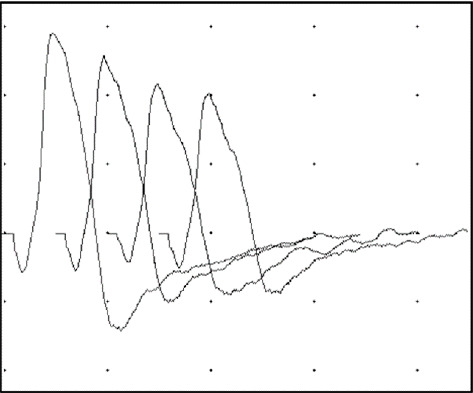
Repetitive stimulation of the right facial nerve at 2 Hz recording from the nasalis muscle 2 min after exercise for 1 min. There is 25% decrement of the compound muscle action potential amplitudes. The calibration marks are 0.2 mV and 10 ms.

After *RAPSN* variants were identified, the patient underwent electromyography (EMG). Repetitive stimulation of the right facial nerve at 2 Hz before and after 30 s of exercise demonstrated a marked decrement response of the compound muscle action potential at rest (25%) which partially corrected after exercise (17%) and worsened 1 min after exercise (28%). These findings are consistent with failure of neuromuscular transmission in the right facial nerve and supports the clinical diagnosis of CMS (Figure 4).

Upon diagnosis, the patient was referred to a neuromuscular clinic where he was evaluated by a pediatric neurologist and was started on treatment with pyridostigmine, an AChE inhibitor. His initial dose was 24 mg four times a day for 3 months and was subsequently increased to 30 mg four times a day, which he tolerated well. The patient experienced improved endurance and higher energy levels with treatment.

## 4. Discussion

CMSs are a heterogeneous group of neuromuscular disorders that are characterized by fatigable or fixed ocular, facial, bulbar, or limb weakness and are caused by variants in genes associated with the NMJ. To date, 35 genes have been associated with CMS [1]. CMSs can be broadly classified based on whether the presynaptic, synaptic, or postsynaptic part of the NMJ is affected, and this distinction has clinical implications because certain CMSs respond to treatment with AChE inhibitors while others are worsened by or unresponsive to AChE inhibitors [1, 4, 12, 14]. AChE inhibitors function to increase the concentration of acetylcholine within the NMJ and thereby increase the likelihood of membrane depolarization [10]. CMS due to biallelic variants in *RAPSN* is caused by dysfunction of the postsynaptic membrane of the NMJ and is responsive to treatment with AChE inhibitors [1, 10, 11]. The primary clinical manifestation of *RAPSN*-associated CMS is fatigable muscle weakness with onset primarily ranging from the neonatal period to age 2 years old; however, there are reports of onset in the later decades of life [15–17].

Here, we present the case of a 4-year-old male with a history of neonatal respiratory distress, feeding difficulty, and hypotonia who has current symptoms of muscle weakness that is exacerbated by acute illness. After extensive nondiagnostic genetic evaluation, trio GS revealed compound heterozygous, biparentally inherited variants in *RAPSN* at the same nucleotide position NM_005055.5:c.264C > A p.(N88K) and NM_005055.5:c.264C > G p.(N88K). His EMG findings confirmed a clinical diagnosis of CMS, and he responded well to treatment with an AChE inhibitor.

This patient's physical exam findings of slight micrognathia, high arched palate, and an elongated face are interesting given that similar findings have been reported in a cohort of patients with variants in the E-box promoter region of *RAPSN* [18–22]. These facial malformations may be secondary to weakness of the facial and masticatory muscles. This case demonstrates that facial malformations can be found in patients with *RAPSN*-associated CMS due to variants outside of the promoter region, thus expanding the genotypic spectrum of facial malformations in *RAPSN*-associated CMS. Other than the *RAPSN* E-box promoter region variants, there has not been a clear genotype-phenotype association described for *RAPSN* variants, which suggests that *RAPSN*-associated CMS has a phenotypic spectrum with variable expressivity [7]. Additional case series in the future may help to clarify if an underlying genotype-phenotype correlation exists.

The patient's maternally inherited variant (c.264C > A) has been reported in the homozygous or compound heterozygous state in several individuals with *RAPSN*-associated CMS (ClinVar ID: 8046) and is classified as likely pathogenic. The c.264C > A encoded p.N88K substitution has been reported in numerous individuals with *RAPSN*-associated CMS [23–26]. The patient's paternally inherited *RAPSN* variant (c.264C > G) has not been reported in ClinVar, is not present in the gnomAD population database, and has not been reported in the literature to date. While the paternally inherited variant lacks additional case data supporting its pathogenicity, given it is predicted to result in the same protein consequence as the established variant p.(N88K), the paternally inherited variant is classified as likely pathogenic. While some case series have observed that individuals who are homozygous for the c.264C > A encoded p.(N88K) change presented with a milder phenotype, other case series have observed a broad range of phenotypes among individuals who are homozygous for the c.264C > A encoded p.(N88K) change [7, 8]. The c.264C > A encoded p.(N88K) change has been shown to affect the interaction of rapsyn with AChR and to significantly reduce the stability of AChR clusters in cellular studies [15].

Interestingly, on initial clinical ES, only the patient's maternally inherited variant (c.264C > A) was reported, which resulted in delayed diagnosis and treatment. One possible explanation for this missed paternal variant is the complexity of annotating and interpreting multiallelic variant sites, which are genomic positions that have been observed to vary to multiple nucleotides [27]. Less than 0.1% of rare variants are predicted to occur at sites that have two alleles that both vary from the reference. However, many of the commonly used variant analysis tools are not equipped to interpret and report multiallelic variant sites [27–29]. At the time of clinical ES in 2019, the clinical laboratory's analysis pipeline was not equipped to detect two different variants at the same position. This case highlights the importance of evaluating for multiallelic sites and improving our clinical sequencing analysis pipelines to ensure that different pathogenic variants at the same nucleotide position are not overlooked.

## Data Availability

Individual level sequencing data are available through dbGAP accession number phs001232 (v5.p2). Additional data are available from the authors upon request.

## Nomenclature

AChE: Acetylcholinesterase
AChR: Acetylcholine receptor
ACMG: American College of Medical Genetics
CMS: Congenital myasthenic syndrome
CPAP: Continuous positive airway pressure
EMG: Electromyography
ES: Exome sequencing
GS: Genome sequencing
MRI: Magnetic resonance imaging
NICU: Neonatal intensive care unit
NMJ: Neuromuscular junction
Rapsyn: Receptor-associated protein of the synapse
UDN: Undiagnosed Diseases Network
